# Crystal structure of (dibenzo-21-crown-7)di­iodido­samarium(II) 1,2-di­meth­oxy­ethane hemisolvate

**DOI:** 10.1107/S2056989026001374

**Published:** 2026-03-17

**Authors:** Hannah B. Wineinger, Joseph M. Sperling, Thomas E. Albrecht

**Affiliations:** a Colorado School of Mines, 1500 Illinois Street Golden CO 80401 USA; University of Missouri-Columbia, USA

**Keywords:** crystal structure, Sm(dibenzo-21-crown-7)I_2_, samarium(II)

## Abstract

The title compound, [SmI_2_(C_22_H_28_O_7_)]·0.5C_4_H_10_O_2_ or Sm(dibenzo-21-crown-7)I_2_·0.5dimethoxyethane, was obtained as a minor product by layering di­meth­oxy­ethane solutions of SmI_2_ and dibenzo-21-crown-7. The asymmetric unit consists of one Sm(dibenzo-21-crown-7)I_2_ moiety and half a di­meth­oxy­ethane solvent mol­ecule in the outer sphere. Of the seven oxygen atoms available for coordination in dibenzo-21-crown-7, only six are coordinated, forming a plane of coordination around samarium(II). The remaining oxygen and its adjacent benzene ring ‘jack knife’ perpendicularly relative to this plane of coordination.

## Abstract 2

The title compound, [SmI_2_(C_22_H_28_O_7_)]·0.5C_4_H_10_O_2_ or Sm(dibenzo-21-crown-7)I_2_, was obtained as a minor product by layering di­meth­oxy­ethane solutions of SmI_2_ and dibenzo-21-crown-7. The asymmetric unit consists of one Sm(dibenzo-21-crown-7)I_2_ moiety and half a di­meth­oxy­ethane solvent mol­ecule in the outer sphere.

## 1. Chemical context

Traditional divalent lanthanides such as Eu^2+^, Yb^2+^, Sm^2+^, and Tm^2+^ are relatively accessible despite being thermodynamically less favorable than their trivalent counterparts (Wedal & Evans, 2021 ▸; Nief, 2010 ▸). Samarium(II) is one of the more challenging traditional divalent lanthanides to stabilize due to its +3/+2 electrochemical potential of −1.55 V, but this can be overcome using ligands that saturate the available coordination sites while avoiding easily reducible functional groups (Wineinger *et al.*, 2025*b* ▸). Crown ether mol­ecules feature variable O-donor atoms without introducing reducible substituents, and such mol­ecules have a demonstrated utility for complexation to samarium(II) in the solution and solid phases (Poe *et al.*, 2021*a* ▸,*b* ▸, 2022 ▸; Starynowicz, 2004 ▸). In fact, there are a number of crystallographic studies focused on finding the best ‘size match’ crown ether for samarium(II) using 12-crown-4 (Wineinger *et al.*, 2024 ▸), (benzo-)15-crown-5 (Poe *et al.*, 2021*b* ▸), (benzo-)18-crown-6 (Poe *et al.*, 2022 ▸), dibenzo-24-crown-8 (Wineinger *et al.*, 2025*a* ▸), and dibenzo-30-crown-10 (White *et al.*, 2019 ▸).
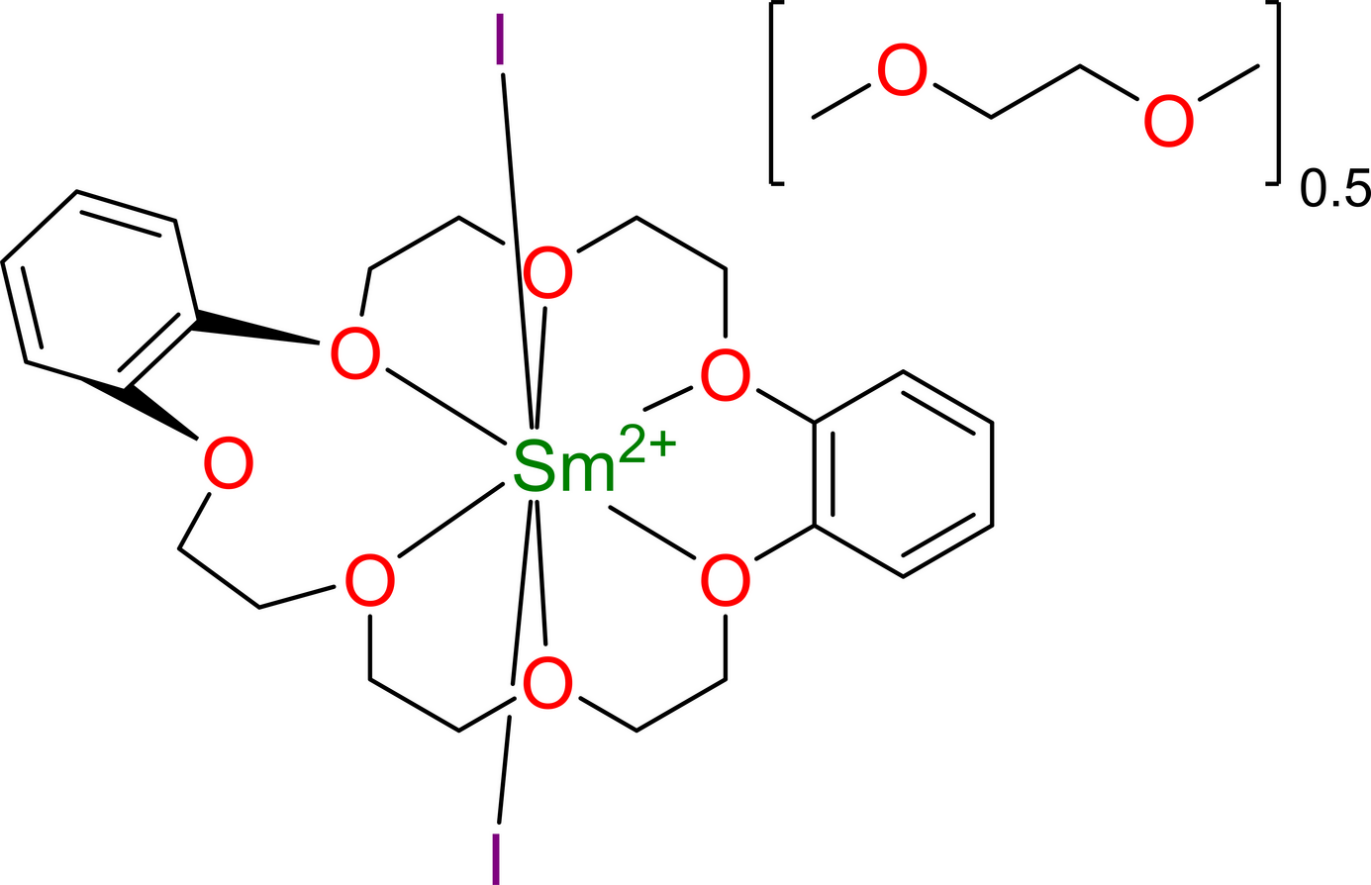


Herein, we report the synthesis and isolation of Sm(db21c7)I_2_·0.5dme (where db21c7 = dibenzo-21-crown-7, dme = 1,2-di­meth­oxy­ethane), a henceforth overlooked crown ether in the study of Sm^2+^/crown ether complexation.

## 2. Structural commentary

**Figure 1:**
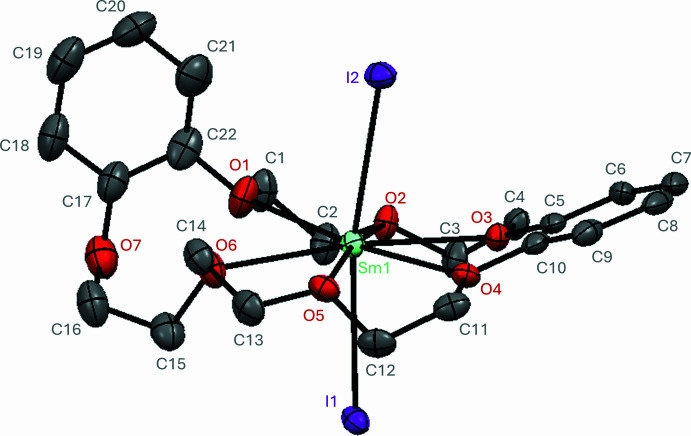
Structure of Sm(db21*c*7)I_2_·0.5dme with displacement ellipsoids drawn at the 50% probability level. H atoms and non-coordinating solvent mol­ecules are omitted for clarity.

Sm(db21c7)I_2_.0.5dme (Fig. 1 ▸) crystallizes in the monoclinic space group *C*2/*c* (No. 15) with one Sm(db21c7)I_2_ mol­ecule and half a dme mol­ecule in the asymmetric unit (Wyckoff position 4*e*, site symmetry 2, found at the mol­ecule’s midpoint). The samarium(II) metal center sits inside the largely planar dibenzo-21-crown-7 mol­ecule, where six of the seven available oxygen atoms are coordinated to the metal center with Sm^2+^—O bond lengths ranging from 2.651 (5) to 2.779 (5) Å. The seventh oxygen atom remains uncoordinated, causing the adjacent benzo substituent to ‘jack-knife’ almost perpendicularly to the rest of the planar-like crown. The remaining 2 coordination sites, above and below the plane of the coordinating crown, are occupied by iodide atoms with an I—Sm^2+^—I angle of 170.18 (2)° and Sm^2+^—I bond lengths of 3.1992 (7) and 3.2711 (7) Å. All torsion angles (O—C—C—O) in the crown ether ethyl­ene chains are approximately gauche [±60 (8)°], and each five-membered chelation ring (ignoring benzo rings) can be assigned as a positive (δ) or negative (λ) torsion angle, allowing a fingerprint assignment of the crown ether conformation. In this case, two enanti­omeric db21c7 conformations are present due to the centrosymmetric space group where the Sm^2+^ center is not located on a Wyckoff position: (λδ)(λδδ) and (δλ)(δλλ). The chirality of the individual Sm(db21c7)I_2_ mol­ecules may have utility towards building nonlinear optical or magnetic materials (Long *et al.*, 2018 ▸).

## 3. Supra­molecular features

**Figure 2:**
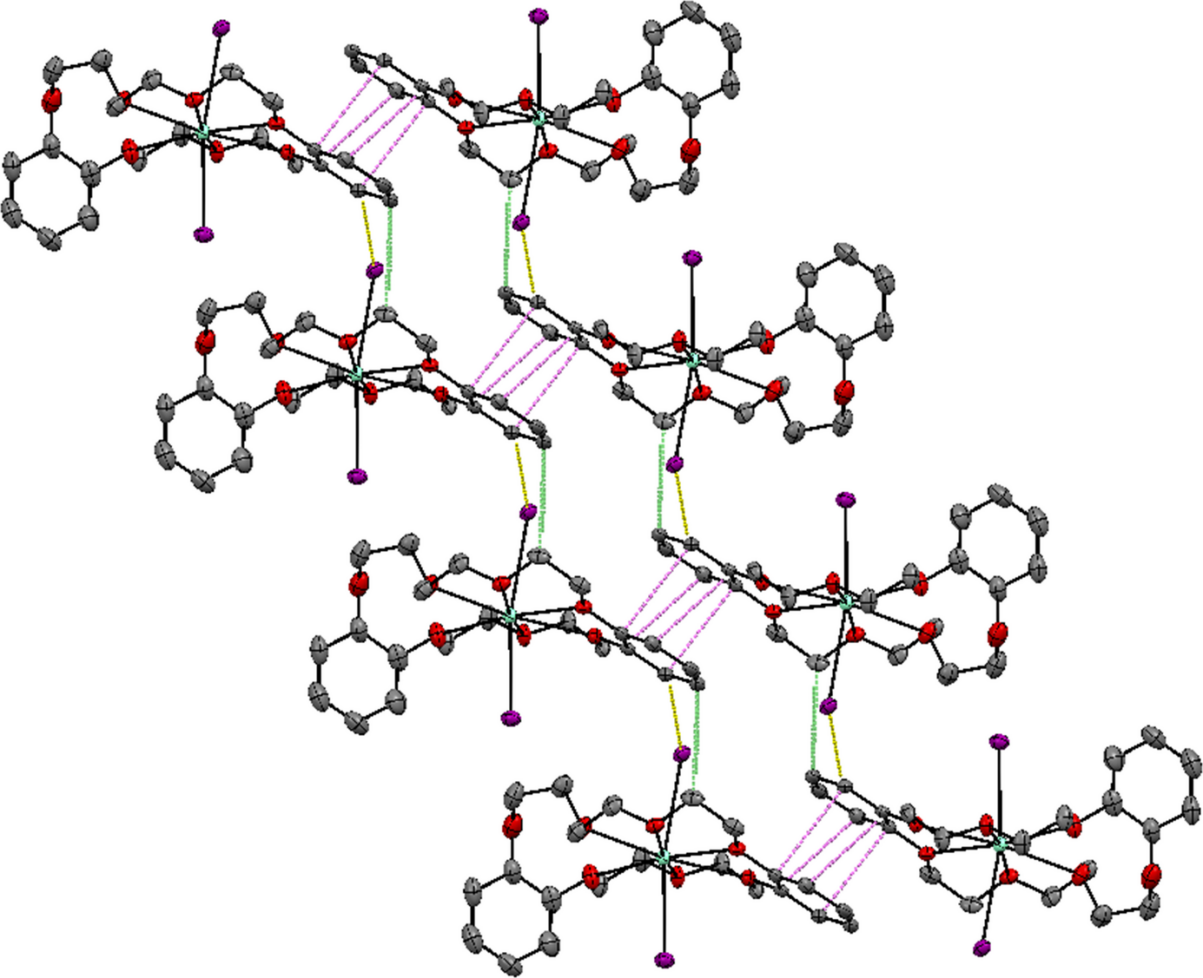
Supra­molecular assembly of Sm(db21*c*7)I_2_·0.5dme, where short contacts between π-stacked benzene rings are shown in violet, C_benzene-_–H⋯I inter­actions in yellow, and C_methyl­ene_—H⋯C_benzene_ inter­actions in green. Displacement ellipsoids are drawn at the 50% probability level, where samarium atoms are represented as lime green, oxygen as red, carbon as gray, and iodide as purple. Hydrogen atoms have been omitted for clarity.

In the crystal, Sm(db21c7)I_2_ units inter­act pairwise through *π*-stacking [centroid–centroid distance of 3.533 (4) Å] of the ‘planar’ oriented benzene rings (Fig. 2 ▸, in violet), and each pair of Sm(db21c7)I_2_ units is linked to the adjacent pairs *via* C_benzene_—H⋯I (yellow) and C_methyl­ene_—H⋯C_benzene_ (green) inter­actions (Table 1 ▸). The ‘jack-knifed’ benzene rings show several short contacts to the nearby dme mol­ecule (C_benzene_—H⋯O/C_dme_) and one inter­action with the non-coordinating db21c7 oxygen of a nearby Sm(db21c7)I_2_ unit (C_benzene_—H⋯O_db21c7_).

## 4. Database survey

**Figure 3:**
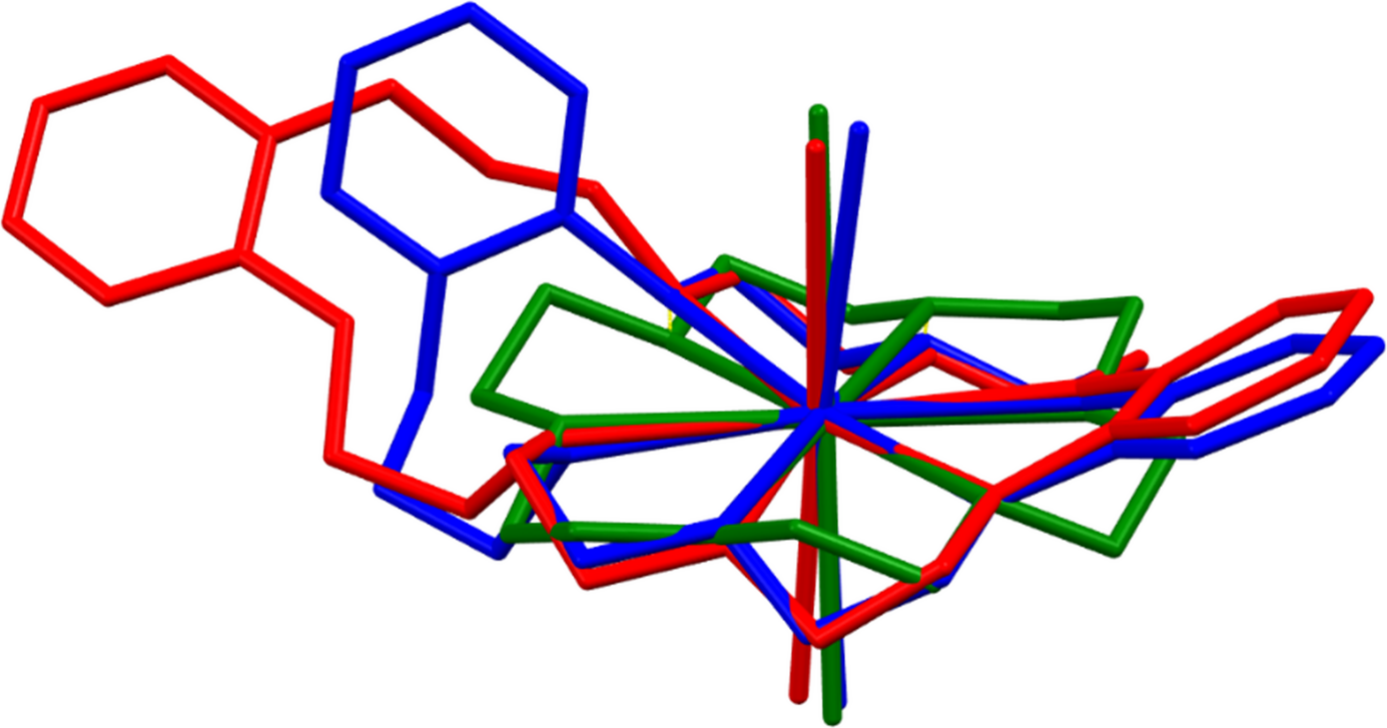
Overlaid structures of Sm(18-crown-6)I_2_ (LARFUJ, green; Poe *et al.*, 2022 ▸), Sm(dibenzo-21-crown-7)I_2_·0.5dme (blue), and Sm(dibenzo-24-crown-8)I_2_ (VUKXEI, red; Wineinger *et al.*, 2025*a* ▸) generated using Mercury (Macrae *et al.*, 2020 ▸).

Metal/(dibenzo-)21-crown-7 coordination complexes are relatively rare, with only four examples found in the CSD (version of November 24, 2025; Groom *et al.*, 2016 ▸): three hepta­dentate Cs^+^/(dibenzo-)21-crown-7 complexes (Yan *et al.*, 2016 ▸; Zhu *et al.*, 2022 ▸) and one tridentate Ag^+^/dibenzo-21-crown-7 (Wen *et al.*, 2002 ▸). Samarium(II) crown ether compounds are more common, where samarium(II) complexation to crown ethers ranging in size from 12-crown-4 to dibenzo-30-crown-10 are known (Poe *et al.*, 2021*a* ▸,*b* ▸, 2022 ▸; Starynowicz, 2004 ▸; Wineinger *et al.*, 2024 ▸, 2025*b* ▸; White *et al.*, 2019 ▸). A comparison of Sm(db21c7)I_2_·0.5dme with Sm(18-crown-6)I_2_ (a smaller crown) and Sm(db24c8)I_2_ (a larger crown) reveals consistent Sm^2+^—O bond lengths [2.651 (5)–2.779 (5) Å; Poe *et al.*, 2022 ▸; Wineinger *et al.*, 2025*a* ▸], where the Sm^2+^ center remains 8-coordinate in spite of the changing cavity size and number of potential coordinating atoms (see Fig. 3 ▸). Between these crowns, dibenzo-21-crown-7 functions as an inter­mediary; it is simultaneously too big to achieve reasonable Sm^2+^—O bond lengths in a hepta­dentate ‘planar’ conformation and too small to contort into a ‘boat-like’ conformation such that all available oxygen atoms are coordinated, such as in [Sm(db24c8)(solvent)_*n*_]^2+^ (solvent = THF, CH_3_CN, dme; *n* = 1, 2).

## 5. Synthesis and crystallization

A solution of db21c7 was layered onto a filtered solution of SmI_2_ in dme, resulting in the formation of bulk red solid and small, blue plate-shaped single crystals of Sm(db21c7)I_2_·0.5dme after several days. The bulk red solid did not form single crystals and was not further characterized.

## 6. Refinement

Crystal data, data collection and structure refinement details are summarized in Table 1 ▸. H atoms were positioned geom­etrically (C—H = 0.95–0.99 Å) and refined as riding with *U*_iso_(H) = 1.2–1.5*U*_eq_(C).

## Supplementary Material

Crystal structure: contains datablock(s) I. DOI: 10.1107/S2056989026001374/ev2025sup1.cif

Structure factors: contains datablock(s) I. DOI: 10.1107/S2056989026001374/ev2025Isup3.hkl

CCDC reference: 2525331

Additional supporting information:  crystallographic information; 3D view; checkCIF report

## Figures and Tables

**Table 1:** Hydrogen-bond geometry (Å, °)

*D*—H⋯*A*	*D*—H	H⋯*A*	*D*⋯*A*	*D*—H⋯*A*
C4—H4*A*⋯C9^i^	0.99	2.86	3.694 (10)	142
C6—H6⋯I1^ii^	0.95	3.12	4.021 (8)	159
C11—H11*A*⋯C9	0.99	2.76	2.797 (11)	82
C12—H12*A*⋯C7^iii^	0.99	2.85	3.379 (11)	114
C19—H19⋯O7^iv^	0.95	2.57	3.474 (13)	160
C20—H20⋯O8	0.95	2.71	3.614 (17)	159
C24—H24*A*⋯C18^ii^	0.99	2.86	3.736 (18)	148

Symmetry codes: (i) 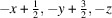; (ii) 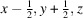; (iii) 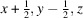; (iv) 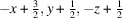.

**Table 2:** Experimental details

Crystal data	
Chemical formula	[SmI_2_(C_22_H_28_O_7_)]·0.5C_4_H_10_O_2_
*M*_r_	853.65
Crystal system, space group	Monoclinic, *C*2/*c*
Temperature (K)	100
*a*, *b*, *c* (Å)	13.4402 (15), 11.3258 (12), 37.515 (4)
β (°)	91.168 (4)
*V* (Å^3^)	5709.5 (11)
*Z*	8
Radiation type	Mo *K*α
μ (mm^−1^)	4.26
Crystal size (mm)	0.14 × 0.07 × 0.06

Data collection	
Diffractometer	Bruker D8 Quest
Absorption correction	Multi-scan (*SADABS*; Krause *et al.*, 2015 ▸)
*T*_min_, *T*_max_	0.557, 0.746
No. of measured, independent and observed [*I* > 2σ(*I*)] reflections	112554, 7097, 6933
*R*_int_	0.056
(sin θ/λ)_max_ (Å^−1^)	0.667

Refinement	
*R*[*F*^2^ > 2σ(*F*^2^)], *wR*(*F*^2^), *S*	0.048, 0.110, 1.37
No. of reflections	7097
No. of parameters	317
H-atom treatment	H-atom parameters constrained
Δρ_max_, Δρ_min_ (e Å^−3^)	1.51, −1.65

Computer programs: *APEX4* (Bruker, 2021 ▸), *SAINT* (Bruker, 2016 ▸), *SHELXS* (Sheldrick, 2008 ▸), *SHELXL2018/3* (Sheldrick, 2015 ▸) and *OLEX2* (Dolomanov *et al.*, 2009 ▸).

## supplementary crystallographic information

### (Dibenzo-21-crown-7)diiodidosamarium(II) 1,2-dimethoxyethane hemisolvate Crystal data

**Table:**

[SmI_2_(C_22_H_28_O_7_)]·0.5C_4_H_10_O_2_	*F*(000) = 3272
*M**_r_* = 853.65	*D*_x_ = 1.986 Mg m^−^^3^
Monoclinic, *C*2/*c*	Mo *K*α radiation, λ = 0.71073 Å
*a* = 13.4402 (15) Å	Cell parameters from 9778 reflections
*b* = 11.3258 (12) Å	θ = 2.4–28.3°
*c* = 37.515 (4) Å	µ = 4.26 mm^−^^1^
β = 91.168 (4)°	*T* = 100 K
*V* = 5709.5 (11) Å^3^	Plate, clear light blue
*Z* = 8	0.14 × 0.07 × 0.06 mm

### (Dibenzo-21-crown-7)diiodidosamarium(II) 1,2-dimethoxyethane hemisolvate Data collection

**Table:**

Bruker D8 Quest diffractometer	7097 independent reflections
Radiation source: sealed tube	6933 reflections with *I* > 2σ(*I*)
Graphite monochromator	*R*_int_ = 0.056
Detector resolution: 8 pixels mm^-1^	θ_max_ = 28.3°, θ_min_ = 2.4°
ω and φ scans	*h* = −17→17
Absorption correction: multi-scan (SADABS; Krause *et al.*, 2015)	*k* = −15→15
*T*_min_ = 0.557, *T*_max_ = 0.746	*l* = −50→50
112554 measured reflections	

### (Dibenzo-21-crown-7)diiodidosamarium(II) 1,2-dimethoxyethane hemisolvate Refinement

**Table:**

Refinement on *F*^2^	Primary atom site location: structure-invariant direct methods
Least-squares matrix: full	Hydrogen site location: inferred from neighbouring sites
*R*[*F*^2^ > 2σ(*F*^2^)] = 0.048	H-atom parameters constrained
*wR*(*F*^2^) = 0.110	*w* = 1/[σ^2^(*F*_o_^2^) + 132.2111*P*] where *P* = (*F*_o_^2^ + 2*F*_c_^2^)/3
*S* = 1.37	(Δ/σ)_max_ = 0.001
7097 reflections	Δρ_max_ = 1.51 e Å^−^^3^
317 parameters	Δρ_min_ = −1.65 e Å^−^^3^
0 restraints	

### (Dibenzo-21-crown-7)diiodidosamarium(II) 1,2-dimethoxyethane hemisolvate Special details

**Table:**

Geometry. All esds (except the esd in the dihedral angle between two l.s. planes) are estimated using the full covariance matrix. The cell esds are taken into account individually in the estimation of esds in distances, angles and torsion angles; correlations between esds in cell parameters are only used when they are defined by crystal symmetry. An approximate (isotropic) treatment of cell esds is used for estimating esds involving l.s. planes.

### (Dibenzo-21-crown-7)diiodidosamarium(II) 1,2-dimethoxyethane hemisolvate Fractional atomic coordinates and isotropic or equivalent isotropic displacement parameters (Å^2^)

**Table:**

	*x*	*y*	*z*	*U*_iso_*/*U*_eq_	
Sm1	0.48892 (2)	0.75926 (3)	0.10265 (2)	0.02064 (8)	
I1	0.47205 (3)	0.47468 (4)	0.08893 (2)	0.02773 (11)	
I2	0.47814 (3)	1.04132 (4)	0.10508 (2)	0.03111 (12)	
O3	0.3077 (3)	0.7793 (4)	0.07158 (12)	0.0227 (9)	
O4	0.4623 (3)	0.7901 (4)	0.03199 (12)	0.0241 (9)	
O2	0.3283 (4)	0.7195 (5)	0.14164 (13)	0.0294 (11)	
O5	0.6453 (4)	0.7644 (4)	0.05986 (13)	0.0271 (10)	
O6	0.6674 (4)	0.7128 (5)	0.13061 (15)	0.0340 (12)	
O1	0.5043 (4)	0.7501 (6)	0.17657 (14)	0.0391 (13)	
C11	0.5442 (6)	0.7810 (7)	0.00813 (19)	0.0304 (15)	
H11A	0.570753	0.860292	0.002561	0.036*	
H11B	0.522729	0.742412	−0.014423	0.036*	
C14	0.7457 (5)	0.7709 (7)	0.1115 (2)	0.0342 (16)	
H14A	0.811160	0.751254	0.122540	0.041*	
H14B	0.736669	0.857600	0.112563	0.041*	
C9	0.3734 (6)	0.9046 (6)	−0.01372 (19)	0.0317 (16)	
H9	0.430442	0.910419	−0.028153	0.038*	
C5	0.2940 (5)	0.8397 (5)	0.04008 (17)	0.0228 (13)	
C4	0.2201 (5)	0.7604 (7)	0.09245 (19)	0.0290 (15)	
H4A	0.164859	0.730011	0.077180	0.035*	
H4B	0.198640	0.835288	0.103511	0.035*	
C8	0.2830 (7)	0.9544 (6)	−0.0251 (2)	0.040 (2)	
H8	0.278774	0.994330	−0.047332	0.048*	
C6	0.2054 (6)	0.8900 (6)	0.0286 (2)	0.0308 (16)	
H6	0.148344	0.886574	0.043068	0.037*	
C13	0.7416 (5)	0.7303 (8)	0.0739 (2)	0.0370 (17)	
H13A	0.795030	0.767879	0.060134	0.044*	
H13B	0.749770	0.643517	0.072687	0.044*	
C7	0.2006 (7)	0.9460 (7)	−0.0045 (2)	0.0372 (19)	
H7	0.139282	0.978598	−0.012842	0.045*	
C1	0.4177 (6)	0.7219 (9)	0.1963 (2)	0.0389 (19)	
H1A	0.383286	0.795189	0.203549	0.047*	
H1B	0.436470	0.677090	0.218082	0.047*	
C10	0.3789 (5)	0.8474 (6)	0.01854 (18)	0.0262 (14)	
C12	0.6213 (6)	0.7084 (7)	0.0270 (2)	0.0325 (16)	
H12A	0.595529	0.627889	0.031389	0.039*	
H12B	0.681474	0.701864	0.012355	0.039*	
C3	0.2477 (5)	0.6720 (8)	0.12058 (19)	0.0315 (16)	
H3A	0.189984	0.655961	0.135812	0.038*	
H3B	0.268239	0.596861	0.109391	0.038*	
C2	0.3504 (6)	0.6489 (8)	0.1727 (2)	0.0378 (18)	
H2A	0.384003	0.574735	0.165806	0.045*	
H2B	0.288497	0.628734	0.185152	0.045*	
C22	0.5734 (6)	0.8198 (8)	0.1963 (2)	0.040 (2)	
C21	0.5588 (8)	0.9330 (10)	0.2028 (2)	0.053 (2)	
H21	0.500032	0.970210	0.193838	0.064*	
C20	0.6286 (8)	0.9991 (9)	0.2226 (3)	0.052 (2)	
H20	0.617964	1.080741	0.226984	0.062*	
C17	0.6593 (7)	0.7605 (9)	0.2099 (2)	0.043 (2)	
C18	0.7295 (7)	0.8246 (10)	0.2294 (2)	0.048 (2)	
H18	0.788536	0.787929	0.238300	0.058*	
O8	0.5493 (13)	1.3036 (12)	0.2171 (3)	0.166 (8)	
O7	0.6700 (5)	0.6392 (6)	0.20610 (18)	0.0486 (16)	
C16	0.7414 (7)	0.6038 (10)	0.1809 (3)	0.050 (2)	
H16A	0.769167	0.526063	0.187885	0.060*	
H16B	0.796759	0.661561	0.180933	0.060*	
C15	0.6962 (7)	0.5954 (8)	0.1436 (2)	0.043 (2)	
H15A	0.745081	0.560134	0.127379	0.052*	
H15B	0.636959	0.543536	0.143934	0.052*	
C19	0.7117 (8)	0.9441 (11)	0.2355 (3)	0.056 (3)	
H19	0.758973	0.988428	0.249096	0.067*	
C23	0.548 (2)	1.2966 (16)	0.1801 (4)	0.169 (12)	
H23A	0.599850	1.347511	0.170611	0.254*	
H23B	0.482685	1.322420	0.170832	0.254*	
H23C	0.559700	1.214748	0.172855	0.254*	
C24	0.5018 (11)	1.3910 (15)	0.2302 (4)	0.094 (5)	
H24A	0.432873	1.390254	0.220398	0.113*	
H24B	0.533542	1.465260	0.222328	0.113*	

### (Dibenzo-21-crown-7)diiodidosamarium(II) 1,2-dimethoxyethane hemisolvate Atomic displacement parameters (Å^2^)

**Table:**

	*U*^11^	*U*^22^	*U*^33^	*U*^12^	*U*^13^	*U*^23^
Sm1	0.01710 (15)	0.02248 (16)	0.02237 (15)	−0.00078 (12)	0.00078 (11)	0.00182 (13)
I1	0.0238 (2)	0.0224 (2)	0.0369 (2)	0.00068 (16)	−0.00101 (17)	0.00491 (18)
I2	0.0270 (2)	0.0247 (2)	0.0416 (3)	−0.00134 (17)	0.00108 (18)	−0.00476 (19)
O3	0.020 (2)	0.026 (2)	0.022 (2)	0.0001 (18)	−0.0010 (17)	0.0003 (18)
O4	0.025 (2)	0.022 (2)	0.025 (2)	0.0022 (18)	0.0029 (18)	−0.0015 (18)
O2	0.023 (2)	0.041 (3)	0.024 (2)	−0.008 (2)	−0.0033 (18)	0.005 (2)
O5	0.024 (2)	0.024 (2)	0.033 (3)	0.0019 (19)	0.0062 (19)	0.002 (2)
O6	0.021 (2)	0.042 (3)	0.039 (3)	0.000 (2)	0.000 (2)	0.011 (2)
O1	0.028 (3)	0.063 (4)	0.026 (3)	−0.009 (3)	−0.001 (2)	−0.003 (3)
C11	0.036 (4)	0.027 (4)	0.029 (3)	−0.009 (3)	0.010 (3)	−0.002 (3)
C14	0.022 (3)	0.037 (4)	0.044 (4)	−0.002 (3)	0.002 (3)	0.008 (3)
C9	0.049 (4)	0.023 (3)	0.022 (3)	−0.005 (3)	−0.004 (3)	0.000 (3)
C5	0.029 (3)	0.015 (3)	0.024 (3)	0.000 (2)	−0.004 (2)	−0.005 (2)
C4	0.018 (3)	0.040 (4)	0.028 (3)	−0.001 (3)	0.001 (2)	−0.007 (3)
C8	0.075 (6)	0.017 (3)	0.027 (4)	0.002 (4)	−0.019 (4)	−0.002 (3)
C6	0.035 (4)	0.024 (3)	0.033 (4)	0.010 (3)	−0.011 (3)	−0.009 (3)
C13	0.020 (3)	0.042 (4)	0.048 (5)	0.001 (3)	0.005 (3)	0.002 (4)
C7	0.052 (5)	0.021 (3)	0.038 (4)	0.009 (3)	−0.021 (4)	−0.008 (3)
C1	0.031 (4)	0.060 (6)	0.026 (4)	−0.004 (4)	0.001 (3)	0.007 (4)
C10	0.036 (4)	0.018 (3)	0.024 (3)	−0.003 (3)	−0.005 (3)	−0.004 (2)
C12	0.031 (4)	0.032 (4)	0.036 (4)	0.000 (3)	0.016 (3)	−0.005 (3)
C3	0.016 (3)	0.048 (5)	0.031 (4)	−0.009 (3)	0.000 (3)	0.003 (3)
C2	0.035 (4)	0.050 (5)	0.028 (4)	−0.011 (4)	−0.001 (3)	0.012 (3)
C22	0.042 (4)	0.051 (5)	0.028 (4)	−0.022 (4)	0.003 (3)	0.011 (4)
C21	0.063 (6)	0.065 (7)	0.032 (4)	−0.001 (5)	−0.001 (4)	−0.002 (4)
C20	0.067 (6)	0.046 (5)	0.043 (5)	−0.015 (5)	0.003 (4)	−0.013 (4)
C17	0.044 (5)	0.053 (5)	0.033 (4)	−0.012 (4)	−0.007 (3)	0.006 (4)
C18	0.037 (5)	0.073 (7)	0.035 (4)	−0.014 (4)	−0.007 (3)	0.006 (4)
O8	0.290 (19)	0.152 (11)	0.054 (6)	0.164 (13)	−0.035 (8)	−0.005 (6)
O7	0.042 (3)	0.051 (4)	0.053 (4)	−0.003 (3)	−0.008 (3)	0.016 (3)
C16	0.036 (5)	0.061 (6)	0.053 (5)	0.000 (4)	−0.007 (4)	0.020 (5)
C15	0.040 (5)	0.038 (5)	0.051 (5)	−0.005 (4)	−0.004 (4)	0.012 (4)
C19	0.051 (6)	0.079 (8)	0.037 (5)	−0.019 (5)	−0.003 (4)	−0.008 (5)
C23	0.39 (4)	0.078 (11)	0.044 (8)	0.037 (17)	−0.009 (14)	−0.004 (8)
C24	0.069 (9)	0.112 (13)	0.102 (11)	0.009 (9)	0.033 (8)	0.032 (9)

### (Dibenzo-21-crown-7)diiodidosamarium(II) 1,2-dimethoxyethane hemisolvate Geometric parameters (Å, º)

**Table:**

Sm1—I1	3.2711 (7)	C13—H13A	0.9900
Sm1—I2	3.1992 (7)	C13—H13B	0.9900
Sm1—O3	2.688 (4)	C7—H7	0.9500
Sm1—O4	2.690 (5)	C1—H1A	0.9900
Sm1—O2	2.671 (5)	C1—H1B	0.9900
Sm1—O5	2.671 (5)	C1—C2	1.502 (11)
Sm1—O6	2.651 (5)	C12—H12A	0.9900
Sm1—O1	2.779 (5)	C12—H12B	0.9900
O3—C5	1.375 (8)	C3—H3A	0.9900
O3—C4	1.443 (8)	C3—H3B	0.9900
O4—C11	1.436 (8)	C2—H2A	0.9900
O4—C10	1.382 (8)	C2—H2B	0.9900
O2—C3	1.432 (8)	C22—C21	1.320 (14)
O2—C2	1.438 (9)	C22—C17	1.422 (13)
O5—C13	1.440 (9)	C21—H21	0.9500
O5—C12	1.416 (9)	C21—C20	1.403 (14)
O6—C14	1.443 (9)	C20—H20	0.9500
O6—C15	1.466 (10)	C20—C19	1.359 (15)
O1—C1	1.429 (9)	C17—C18	1.386 (12)
O1—C22	1.416 (10)	C17—O7	1.389 (12)
C11—H11A	0.9900	C18—H18	0.9500
C11—H11B	0.9900	C18—C19	1.395 (16)
C11—C12	1.492 (11)	O8—C23	1.388 (16)
C14—H14A	0.9900	O8—C24	1.282 (17)
C14—H14B	0.9900	O7—C16	1.420 (12)
C14—C13	1.485 (12)	C16—H16A	0.9900
C9—H9	0.9500	C16—H16B	0.9900
C9—C8	1.398 (12)	C16—C15	1.515 (13)
C9—C10	1.374 (10)	C15—H15A	0.9900
C5—C6	1.381 (9)	C15—H15B	0.9900
C5—C10	1.414 (10)	C19—H19	0.9500
C4—H4A	0.9900	C23—H23A	0.9800
C4—H4B	0.9900	C23—H23B	0.9800
C4—C3	1.496 (11)	C23—H23C	0.9800
C8—H8	0.9500	C24—C24^i^	1.49 (3)
C8—C7	1.366 (13)	C24—H24A	0.9900
C6—H6	0.9500	C24—H24B	0.9900
C6—C7	1.394 (11)		
			
I2—Sm1—I1	170.178 (17)	O5—C13—H13B	110.5
O3—Sm1—I1	87.45 (10)	C14—C13—H13A	110.5
O3—Sm1—I2	83.51 (10)	C14—C13—H13B	110.5
O3—Sm1—O4	57.38 (14)	H13A—C13—H13B	108.7
O3—Sm1—O1	118.95 (15)	C8—C7—C6	120.8 (7)
O4—Sm1—I1	88.02 (10)	C8—C7—H7	119.6
O4—Sm1—I2	83.87 (10)	C6—C7—H7	119.6
O4—Sm1—O1	173.68 (17)	O1—C1—H1A	110.2
O2—Sm1—I1	82.28 (12)	O1—C1—H1B	110.2
O2—Sm1—I2	96.62 (12)	O1—C1—C2	107.7 (6)
O2—Sm1—O3	61.07 (14)	H1A—C1—H1B	108.5
O2—Sm1—O4	117.96 (14)	C2—C1—H1A	110.2
O2—Sm1—O5	170.96 (16)	C2—C1—H1B	110.2
O2—Sm1—O1	59.44 (15)	O4—C10—C5	114.9 (6)
O5—Sm1—I1	88.85 (11)	C9—C10—O4	124.9 (7)
O5—Sm1—I2	91.83 (11)	C9—C10—C5	120.2 (7)
O5—Sm1—O3	117.04 (15)	O5—C12—C11	108.0 (6)
O5—Sm1—O4	59.69 (15)	O5—C12—H12A	110.1
O5—Sm1—O1	123.87 (15)	O5—C12—H12B	110.1
O6—Sm1—I1	85.76 (13)	C11—C12—H12A	110.1
O6—Sm1—I2	103.17 (13)	C11—C12—H12B	110.1
O6—Sm1—O3	173.12 (17)	H12A—C12—H12B	108.4
O6—Sm1—O4	121.11 (15)	O2—C3—C4	108.2 (6)
O6—Sm1—O2	118.99 (15)	O2—C3—H3A	110.1
O6—Sm1—O5	61.68 (16)	O2—C3—H3B	110.1
O6—Sm1—O1	63.21 (16)	C4—C3—H3A	110.1
O1—Sm1—I1	97.11 (14)	C4—C3—H3B	110.1
O1—Sm1—I2	90.65 (14)	H3A—C3—H3B	108.4
C5—O3—Sm1	121.2 (4)	O2—C2—C1	106.5 (7)
C5—O3—C4	116.3 (5)	O2—C2—H2A	110.4
C4—O3—Sm1	119.6 (4)	O2—C2—H2B	110.4
C11—O4—Sm1	121.1 (4)	C1—C2—H2A	110.4
C10—O4—Sm1	120.7 (4)	C1—C2—H2B	110.4
C10—O4—C11	115.6 (5)	H2A—C2—H2B	108.6
C3—O2—Sm1	111.8 (4)	O1—C22—C17	116.4 (8)
C3—O2—C2	112.3 (6)	C21—C22—O1	122.6 (9)
C2—O2—Sm1	112.4 (4)	C21—C22—C17	121.0 (9)
C13—O5—Sm1	119.1 (4)	C22—C21—H21	119.5
C12—O5—Sm1	110.2 (4)	C22—C21—C20	121.0 (10)
C12—O5—C13	112.6 (6)	C20—C21—H21	119.5
C14—O6—Sm1	112.1 (4)	C21—C20—H20	120.7
C14—O6—C15	112.8 (6)	C19—C20—C21	118.7 (10)
C15—O6—Sm1	122.9 (5)	C19—C20—H20	120.7
C1—O1—Sm1	118.8 (4)	C18—C17—C22	118.8 (9)
C22—O1—Sm1	122.4 (4)	C18—C17—O7	120.1 (9)
C22—O1—C1	112.7 (6)	O7—C17—C22	120.9 (8)
O4—C11—H11A	110.5	C17—C18—H18	120.7
O4—C11—H11B	110.5	C17—C18—C19	118.5 (9)
O4—C11—C12	106.1 (6)	C19—C18—H18	120.7
H11A—C11—H11B	108.7	C24—O8—C23	115.5 (13)
C12—C11—H11A	110.5	C17—O7—C16	114.9 (7)
C12—C11—H11B	110.5	O7—C16—H16A	109.3
O6—C14—H14A	110.0	O7—C16—H16B	109.3
O6—C14—H14B	110.0	O7—C16—C15	111.6 (7)
O6—C14—C13	108.5 (6)	H16A—C16—H16B	108.0
H14A—C14—H14B	108.4	C15—C16—H16A	109.3
C13—C14—H14A	110.0	C15—C16—H16B	109.3
C13—C14—H14B	110.0	O6—C15—C16	110.4 (8)
C8—C9—H9	120.3	O6—C15—H15A	109.6
C10—C9—H9	120.3	O6—C15—H15B	109.6
C10—C9—C8	119.3 (8)	C16—C15—H15A	109.6
O3—C5—C6	125.0 (7)	C16—C15—H15B	109.6
O3—C5—C10	115.2 (6)	H15A—C15—H15B	108.1
C6—C5—C10	119.8 (7)	C20—C19—C18	121.9 (9)
O3—C4—H4A	110.4	C20—C19—H19	119.0
O3—C4—H4B	110.4	C18—C19—H19	119.0
O3—C4—C3	106.7 (5)	O8—C23—H23A	109.5
H4A—C4—H4B	108.6	O8—C23—H23B	109.5
C3—C4—H4A	110.4	O8—C23—H23C	109.5
C3—C4—H4B	110.4	H23A—C23—H23B	109.5
C9—C8—H8	119.7	H23A—C23—H23C	109.5
C7—C8—C9	120.6 (7)	H23B—C23—H23C	109.5
C7—C8—H8	119.7	O8—C24—C24^i^	114.2 (12)
C5—C6—H6	120.3	O8—C24—H24A	108.7
C5—C6—C7	119.3 (8)	O8—C24—H24B	108.7
C7—C6—H6	120.3	C24^i^—C24—H24A	108.7
O5—C13—C14	106.3 (6)	C24^i^—C24—H24B	108.7
O5—C13—H13A	110.5	H24A—C24—H24B	107.6
			
Sm1—O3—C5—C6	−156.1 (5)	C4—O3—C5—C6	4.9 (9)
Sm1—O3—C5—C10	24.7 (7)	C4—O3—C5—C10	−174.3 (6)
Sm1—O3—C4—C3	−30.9 (7)	C8—C9—C10—O4	176.9 (6)
Sm1—O4—C11—C12	22.4 (7)	C8—C9—C10—C5	−0.5 (10)
Sm1—O4—C10—C9	155.3 (5)	C6—C5—C10—O4	−177.6 (6)
Sm1—O4—C10—C5	−27.2 (7)	C6—C5—C10—C9	0.0 (10)
Sm1—O2—C3—C4	−61.3 (6)	C13—O5—C12—C11	−158.0 (6)
Sm1—O2—C2—C1	66.0 (7)	C1—O1—C22—C21	−74.2 (10)
Sm1—O5—C13—C14	−35.1 (8)	C1—O1—C22—C17	104.1 (9)
Sm1—O5—C12—C11	66.5 (6)	C10—O4—C11—C12	−175.5 (6)
Sm1—O6—C14—C13	−59.2 (7)	C10—C9—C8—C7	−0.2 (11)
Sm1—O6—C15—C16	−130.0 (6)	C10—C5—C6—C7	1.1 (10)
Sm1—O1—C1—C2	26.7 (9)	C12—O5—C13—C14	−166.3 (6)
Sm1—O1—C22—C21	77.8 (10)	C3—O2—C2—C1	−166.9 (6)
Sm1—O1—C22—C17	−103.9 (7)	C2—O2—C3—C4	171.3 (6)
O3—C5—C6—C7	−178.0 (6)	C22—O1—C1—C2	179.7 (7)
O3—C5—C10—O4	1.6 (8)	C22—C21—C20—C19	−0.6 (15)
O3—C5—C10—C9	179.3 (6)	C22—C17—C18—C19	1.6 (13)
O3—C4—C3—O2	59.5 (7)	C22—C17—O7—C16	107.4 (9)
O4—C11—C12—O5	−57.1 (7)	C21—C22—C17—C18	−1.9 (13)
O6—C14—C13—O5	60.8 (8)	C21—C22—C17—O7	173.7 (8)
O1—C1—C2—O2	−58.9 (9)	C21—C20—C19—C18	0.4 (15)
O1—C22—C21—C20	179.6 (8)	C17—C22—C21—C20	1.4 (14)
O1—C22—C17—C18	179.8 (7)	C17—C18—C19—C20	−0.9 (15)
O1—C22—C17—O7	−4.6 (11)	C17—O7—C16—C15	−87.5 (10)
C11—O4—C10—C9	−6.9 (9)	C18—C17—O7—C16	−77.0 (10)
C11—O4—C10—C5	170.7 (6)	O7—C17—C18—C19	−174.0 (8)
C14—O6—C15—C16	90.7 (8)	O7—C16—C15—O6	67.0 (10)
C9—C8—C7—C6	1.4 (11)	C15—O6—C14—C13	84.6 (8)
C5—O3—C4—C3	167.8 (6)	C23—O8—C24—C24^i^	175.5 (19)
C5—C6—C7—C8	−1.9 (11)		

Symmetry code: (i) −*x*+1, *y*, −*z*+1/2.

### (Dibenzo-21-crown-7)diiodidosamarium(II) 1,2-dimethoxyethane hemisolvate Hydrogen-bond geometry (Å, º)

**Table:**

*D*—H···*A*	*D*—H	H···*A*	*D*···*A*	*D*—H···*A*
C4—H4*A*···C9^ii^	0.99	2.86	3.694 (10)	142
C6—H6···I1^iii^	0.95	3.12	4.021 (8)	159
C11—H11*A*···C9	0.99	2.76	2.797 (11)	82
C12—H12*A*···C7^iv^	0.99	2.85	3.379 (11)	114
C19—H19···O7^v^	0.95	2.57	3.474 (13)	160
C20—H20···O8	0.95	2.71	3.614 (17)	159
C24—H24*A*···C18^iii^	0.99	2.86	3.736 (18)	148

Symmetry codes: (ii) −*x*+1/2, −*y*+3/2, −*z*; (iii) *x*−1/2, *y*+1/2, *z*; (iv) *x*+1/2, *y*−1/2, *z*; (v) −*x*+3/2, *y*+1/2, −*z*+1/2.
